# Do Antibodies to Malaria Surface Antigens Play a Role in Protecting Mothers From Maternal Anemia?

**DOI:** 10.3389/fimmu.2020.609957

**Published:** 2020-12-18

**Authors:** Madeleine C. Wiebe, Stephanie K. Yanow

**Affiliations:** ^1^ Department of Medical Microbiology and Immunology, University of Alberta, Edmonton, AB, Canada; ^2^ School of Public Health, University of Alberta, Edmonton, AB, Canada

**Keywords:** pregnancy-associated malaria, anemia, inflammation, placenta, VAR2CSA antibodies, VSA_PAM_ antibodies

## Abstract

Pregnancy-associated malaria (PAM) caused by *Plasmodium falciparum* can result in detrimental outcomes for both mother and infant, including low infant birth weight, preterm birth, maternal anemia, spontaneous abortion, and maternal and/or infant mortality. Maternal anemia is a particularly complex outcome, as the body must both maintain erythropoiesis and tolerance of the growing fetus, while directing a Th1 response against the parasite. Underlying the pathogenesis of PAM is the expression of variant surface antigens (VSA_PAM_) on the surface of infected red blood cells (iRBC) that mediate sequestration of the iRBC in the placenta. Naturally acquired antibodies to VSA_PAM_ can block sequestration and activate opsonic phagocytosis, both associated with improved pregnancy outcomes. In this review, we ask whether VSA_PAM_ antibodies can also protect mothers against malarial anemia. Studies were identified where VSA_PAM_ antibody titres and/or function were associated with higher maternal hemoglobin levels, thus supporting additional protective mechanisms for these antibodies against PAM. Yet these associations were not widely observed, and many studies reported no association between protection from maternal anemia and VSA_PAM_ antibodies. We discuss the epidemiological, biological and technical factors that may explain some of the variability among these studies. We appraise the current evidence of these complex interactions between PAM-specific immunity and maternal anemia, propose potential mechanisms, and discuss knowledge gaps.

## Introduction

Pregnant women are especially vulnerable to malaria compared to non-pregnant adults in Africa (1) and 11 million pregnancies are at risk for complications from pregnancy-associated malaria (PAM) on this continent (2) in addition to those in lower transmission regions elsewhere (3). PAM is attributed to poor fetal outcomes including stillbirths (4), low birth weight infants (2) and premature births (5). PAM also significantly contributes to severe maternal anemia (6).

During a *Plasmodium falciparum* infection in high transmission settings, primigravid women have an especially high risk of poor pregnancy outcomes, and this risk decreases with gravidity [reviewed in (7)]. Pregnancy outcomes can affect both mother (maternal anemia, maternal morbidity and mortality) and child (low infant birth weight, pre-term birth, and spontaneous abortion). After several instances of PAM, protection may be conferred by acquired immunity (8). Specifically, antibodies to pregnancy-specific variant surface antigens (VSA_PAM_) expressed on the surface of the infected red blood cell (iRBC) are known to disrupt the adherence of iRBCs to chondroitin sulfate A (CSA) in the placenta and prevent parasite sequestration here (9, 10). One of the key targets of these maternal antibodies is VAR2CSA, a VSA expressed on the iRBC surface that directly binds to CSA (11). VAR2CSA antibodies are associated with improved pregnancy outcomes (12) and as a result, are the focus of vaccines to prevent PAM (13, 14).

While antibody-mediated immune mechanisms are associated with improved birth outcomes, particularly increased infant birth weight, we do not know whether these humoral mechanisms could protect women from developing anemia. By preventing sequestration of iRBCs in the placenta or opsonizing VSAs on the surface of iRBCs, VSA_PAM_ antibodies may promote iRBC phagocytosis in the intervillous spaces (IVS) of the placenta or parasite killing in the spleen, lowering parasitemia and reducing the risk of maternal anemia. However, the development of anemia in PAM and its connection to humoral immunity is not well defined and potential underlying mechanisms of protection warrant investigation (15). Here, we first consider the causes of maternal anemia in PAM, and then examine the studies that investigated associations between anemia and VSA_PAM_ antibodies.

## Mechanisms of Anemia in The Context of PAM

Maternal anemia is a common, but particularly complex consequence of PAM, typically defined as a hemoglobin level less than 11 g/dL, with mild and severe anemia for pregnant women defined at 10–10.9 g/dL and less than 7 g/dL, respectively (16). In a healthy pregnancy, the maternal vasculature must vasodilate approximately 5 weeks into pregnancy, with total blood volume and red cell mass significantly increased (17). An increase in plasma volume compared to red cell production results in physiological anemia during pregnancy that usually resolves by the third trimester (reviewed in (18, 19)). However, anemia is exacerbated by poor nutrition, iron deficiency, and maternal hypertension (reviewed in (20)).

If anemia develops due to malaria infection, this can result in maternal morbidity and mortality as the red cell population is compromised. Some mechanisms of anemia are shared between uncomplicated malaria and PAM, such as obligate hemolysis as the schizont ruptures, which triggers a pro-inflammatory response. In uncomplicated malaria, a profound loss of nRBCs contributes to anemia (21), as red cell deformability is reduced, which has been correlated with a lower hemoglobin concentration (22, 23) but whether this also occurs in the placenta is not known. This loss of non-infected RBCs (nRBCs) is yet to be observed in the PAM setting. Dysregulated erythropoiesis is also reported in uncomplicated malaria, often due to production of pro-inflammatory mediators including cytokines, nitric oxide, and lipoperoxides that can lead to bone marrow dysfunction [reviewed in (24, 25)].

In pregnancy, tolerance to the fetus must be maintained primarily with a T helper (Th) 2 response, through secretions of TGF-β, IL-4, and IL-6 (26–28). However, a *P. falciparum* infection triggers a Th1 response, increasing pro-inflammatory cytokine secretions including IL-1β, TNFα, and IFNγ. Moore et al. observed that intervillous blood mononuclear cells from multigravid women negative for placental malaria had the highest secretions of IFNγ, while cells from primigravid and secundigravid women positive for placental malaria had low IFNγ secretions (29). Thus, an increased production of IFNγ may help control a placental infection. However, pro-inflammatory mediators have also been associated with detrimental placental inflammation. Increased TNFα in primigravid women was associated with placental lesions, while increased IL-1β was associated with congenital malaria (30). This pro-inflammatory response was associated with severe maternal anemia (27). The increase in pro-inflammatory cytokines may also inhibit erythropoiesis in the bone marrow, leading to bone marrow dysfunction and contributing to overall maternal anemia (reviewed in (31), though this has not been shown specifically for PAM. In response to the pro-inflammatory state in the placenta, IL-10 is frequently elevated along with pro-inflammatory mediators in PAM (32, 33) in attempts to maintain a healthy pregnancy (reviewed in (34). Thus, IL-10 can be considered a marker for dangerous placental inflammation (29, 32, 35, 36) and levels of IL-10 correlated with maternal anemia (32, 33).

**Figure 1:**
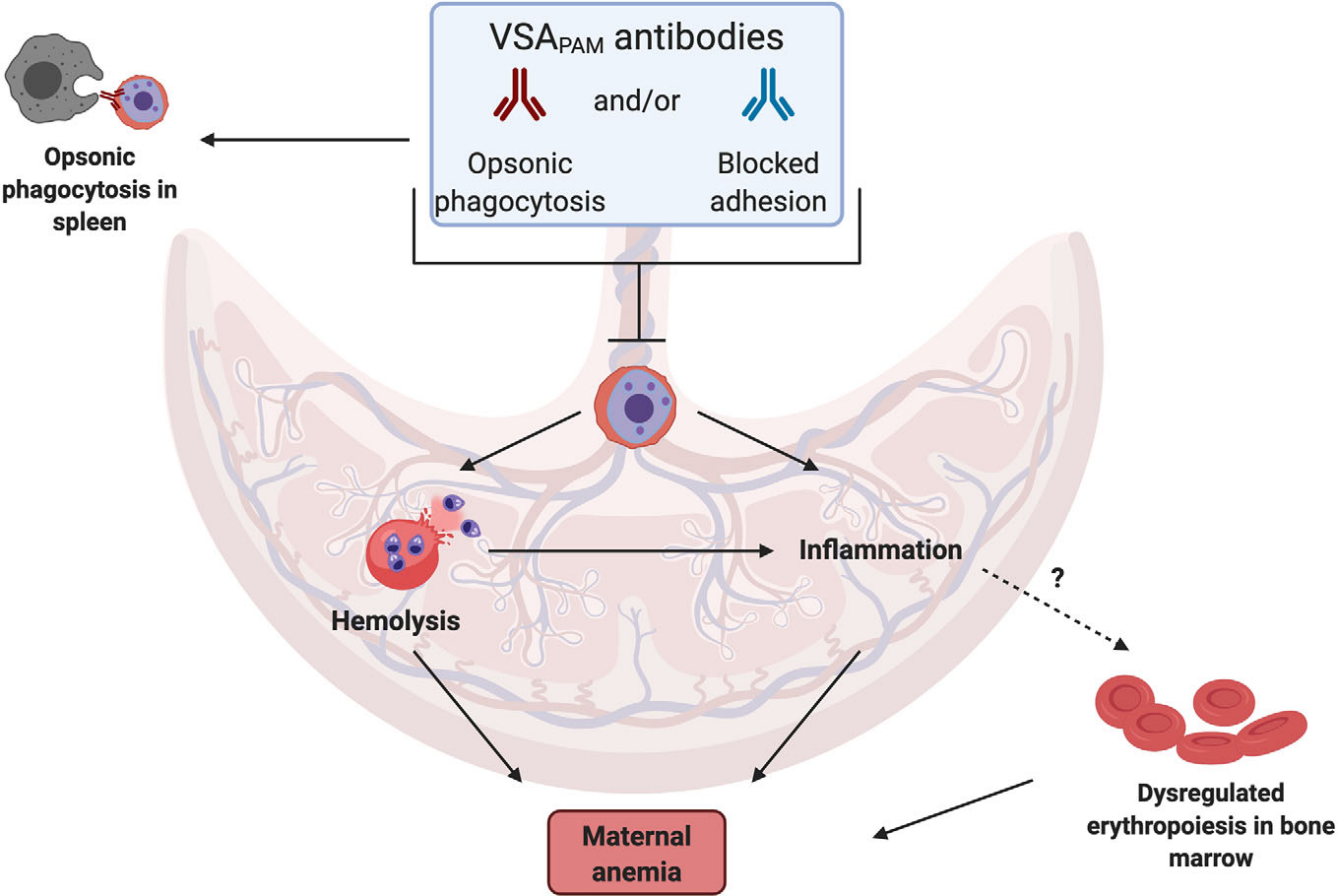
Mechanisms by which VSA_PAM_ antibodies could protect against anemia in PAM. VSA_PAM_ antibodies that bind to the surface of iRBCs can promote iRBC clearance from the placenta by preventing sequestration and/or promoting opsonic phagocytosis in the IVS of the placenta or in the spleen. Both of these mechanisms would reduce placental parasitemia and could prevent several of the downstream mechanisms that lead to maternal anemia; one of these is iRBC hemolysis, where parasite material is released and detected by toll-like receptors present on cells in the IVS, activating a pro-inflammatory response. Reduced inflammation may in turn prevent dysregulated erythropoiesis in the bone marrow, further protecting against maternal anemia. (Created with BioRender.com).

A source of these cytokines in the placenta are monocytes and macrophages (34, 37, 38), which often accumulate in the IVS during infection. Antibodies may fine-tune this response by directly targeting the parasite, blocking iRBC sequestration in the placenta and/or increasing opsonic phagocytosis by targeting iRBCs for engulfment. These potential mechanisms, as illustrated in **Figure 1**, may lower parasitemia in the placenta so that a prolonged inflammatory response is not required.

Further investigation is required to elucidate the mechanisms of anemia in PAM compared with uncomplicated malaria. Far less is published on anemia in PAM, and yet there may be significant differences due to changes in immune regulation that occur in pregnancy. For example, a Th1 response to combat a malaria infection could pose a greater risk in pregnancy compared to uncomplicated malaria as maintenance of a Th2 response is important for the health of the fetus. The investigation of the role of VSA_PAM_ antibodies is further complicated by the multifactorial causes of anemia during PAM, which may alter the relationship between antibody titre/function and maternal anemia. Thus, a connection between VSA_PAM_ antibodies and maternal anemia may be difficult to elucidate.

## Studies of VSA_PAM_ Antibodies and Maternal Anemia

Women without prior exposure to PAM lack antibodies to the unique pregnancy-specific VSA that mediate sequestration of iRBCs to the placenta. Pregnant women can eventually acquire antibodies against these VSA and the levels of these antibodies increase with gravidity (8, 39). There is evidence for an association between VSA_PAM_ antibodies and improved birth weight (40), but less is known whether these antibodies can also protect women from maternal anemia. Here, we discuss the limited studies on VSA_PAM_ antibodies and anemia (**Table 1**).

**Table 1:** Summary of studies on VSA_PAM_ antibodies and maternal anemia.

Citation	Setting	Assays used	Time of serum/plasma collection	Anemia-related outcomes
**No association between VSA_PAM_ antibodies and maternal anemia**				
Duffy and Fried (40)	Kenya	IBA	Delivery	VSA_PAM_ blocking antibodies were not associated with maternal hemoglobin at delivery
Ndam et al. (41)	Benin	ELISA, IBA	Delivery	VSA_PAM_ blocking antibodies were not associated with maternal anemia at delivery
Ataide et al. (42)	Malawi	Flow, opsonic phagocytosis	Late third trimester	VSA_PAM_ antibodies from primigravid women were not associated with maternal hemoglobin
Ataide et al. (43)	Malawi	Flow, opsonic phagocytosis	Late third trimester	VSA_PAM_ antibodies from secundigravid women were not associated with maternal hemoglobin
Mayor et al. (44)	Mozambique	ELISA, Flow	Delivery	VSA_PAM_ antibodies were not associated with maternal anemia
Ndam et al., 2006 (45)	Senegal	ELISA	First to second trimester and delivery	Seroreactive antibodies against VAR2CSA subdomains were not associated with maternal anemia
Serra-Casas et al. (46)	Mozambique	Flow	Delivery	VSA_PAM_ antibodies were not associated with maternal anemia
Aitken et al. (47)	Malawi	Flow	28–34 weeks gestation	VSA_PAM_ antibodies were not associated with maternal hemoglobin
Fried et al. (48)	Mali	Multiplex	Second trimester	Seroreactive antibodies against VAR2CSA subdomains were not correlated with a reduced risk of maternal anemia
**Mixed association between VSA_PAM_ antibodies and maternal anemia**				
Lloyd et al. (49)	Cameroon	Multiplex	Delivery	Seroreactive antibodies against VAR2CSA were associated with lower hematocrit levels and increased prevalence of anemia in women negative for placental infection
Staalsoe et al. (50)	Kenya	Flow	Delivery	Levels of VSA_PAM_ antibodies correlated with hemoglobin in women with chronic placental infection but not acute or past infection
**Association between VSA_PAM_ antibodies and maternal anemia**				
Feng et al., (51)	Malawi	Opsonic phagocytosis	14–20 weeks gestation	Two-fold increase in opsonic phagocytosis index was associated with reduced odds of maternal anemia
Jaworowski et al. (52)	Malawi	Opsonic phagocytosis	Third trimester	VSA_PAM_ antibodies from anemic women displayed significantly lower opsonizing activity compared to non-anemic women
Chandrasiri et al. (53)	Malawi	Flow, opsonic phagocytosis	Before 20 weeks gestation	10% increase in opsonic phagocytosis index was associated with maternal hemoglobin increase of 0.4 g/L at 36 weeks gestation
Sander et al. (54)	Cameroon	Detection of *var2csa* copy number	First, second, and third trimester	In Ngali II, increasing *var2csa* copy number, which correlated with antibodies against DBL4ϵ, was associated with higher maternal hemoglobin
Chandrasiri et al. (55)	Sudan	Flow, opsonic phagocytosis, cytokine profiles	Second and third trimester	DBL5ϵ antibodies and maternal hemoglobin were negatively correlated with pro-inflammatory cytokines; opsonizing antibodies were positively associated with hemoglobin
Gavina et al. (56)	Colombia	ELISA, IBA	First and second trimester	VSA_PAM_ blocking antibodies were positively associated with maternal hemoglobin

### Studies With No Correlation Between VSA_PAM_ Antibodies and Maternal Anemia

In the first study to relate measures of VSA_PAM_ antibodies to birth outcomes, plasma from Kenyan pregnant mothers was tested in the inhibition of binding assay (IBA) with placental isolates and correlated to infant birth weight, gestational age, and maternal hemoglobin at delivery (40). Plasma that blocked >35% of parasite binding to CSA was considered positive for blocking antibodies. Such antibodies were only observed in 1 of 47 primigravid women, while in secondigravid women, anti-adhesion activity was greater and associated with both increased infant birth weight and gestational age. However, there was no relationship between anti-adhesion antibodies and maternal hemoglobin levels (40).

This association was also observed in a study in Benin, where antibody titres were measured against the recombinant proteins DBL1-DBL2X, DBL5ϵ, and DBL6ϵ by ELISA, and functional activity was assessed by IBA (41). Increased anti-adhesion activity was associated with a decreased risk of placental infection at delivery and low birth weight, but there was no association between antibody titres or anti-adhesion activity and maternal anemia at delivery (41).

Opsonic phagocytosis of iRBCs is an important measure of antibody function. In primigravid women from Malawi, serum samples were collected in the late third trimester and tested for reactivity to CS2 parasites by flow cytometry and opsonic phagocytosis (42). No association was observed with maternal anemia or infant birth weight and VSA_PAM_ antibodies. The study was continued with third trimester samples from secundigravid women (43). Again, no association was observed between maternal anemia and VSA_PAM_ IgG or opsonic activity, but a positive correlation between opsonic phagocytosis and infant birth weight was observed.

Other studies compared hemoglobin measurements to IgG titres but not to antibody function. In Mozambique, serum samples collected at delivery were tested by ELISA for reactivity against a number of malarial antigens expressed in uncomplicated malaria and during PAM, including DBL5ϵ and DBL6ϵ, along with reactivity to two placental isolates (44). VSA_PAM_ IgG against one isolate was associated with increased birth weight and gestational age of infants, but no association was found with maternal anemia. In Senegal, IgG was measured against the VAR2CSA subdomains DBL1X, DBL5ϵ, and DBL6ϵ by ELISA and compared with maternal anemia at enrollment and delivery in samples from primigravid, secondigravid, and multigravid women (45). IgG levels against DBL5ϵ and DBL6ϵ at enrollment and delivery were parity-dependent, and high IgG titres correlated with past malaria infection rather than acute PAM. In this study, there was no association between the levels of antibodies against these VAR2CSA domains and maternal anemia or birth weight at any measured time point. Similarly, in a study from Mozambique, the levels of antibody that stained the native VSA on CS2 parasites in flow cytometry did not associate with maternal anemia at delivery (46). Surprisingly, these antibodies did not correlate with other birth outcomes either, including preterm birth or low birth weight.

Two longitudinal studies reported VSA_PAM_ antibody and hemoglobin levels throughout pregnancy, and both failed to identify a correlation between antibody levels at enrollment and pregnancy outcomes. The first study reported IgG titres in pregnant Malawi women from serum collected at various points in pregnancy including study enrollment, 28–34 gestational weeks, and 1–6 months postpartum (47). There was a non-significant downward trend in antibody levels against VSA-expressing parasites CS2 and HCS3-VSA from enrollment to one month postpartum, but IgG remained detectable 6 months after delivery in 72% of women. Despite the persistence of the VSA_PAM_ antibodies, they were not associated with an increase in maternal hemoglobin levels. Fried et al. published findings from Mali with plasma samples collected at enrollment, 30–32 gestational weeks, and at delivery (48). A multiplex bead assay was used to determine seropositivity to a large number of VAR2CSA subdomains, including DBL2X, DBL4ϵ, and ID1-ID2a, but notably not the full-length protein. Increased antibody titres against either of the DBL domains were not significantly associated with any type of protection.

### Mixed Association Between VSA_PAM_ Antibodies and Maternal Anemia

In Cameroon, two groups of women, one positive and one negative for placental malaria, both had overall low antibody levels to full-length VAR2CSA at delivery (49). Among women positive for placental malaria, higher seropositivity against VAR2CSA was associated with a reduced risk of placental parasitemia and low birth weight infants, but not maternal anemia at delivery. Interestingly, in women negative for placental malaria, the presence of VAR2CSA antibodies was associated with lower hematocrit at delivery and increased prevalence of maternal anemia. This is hypothesized to be the product of a malaria infection that was cleared early in pregnancy, with the presence of antibodies marking this past infection. But, the maternal hematocrit and prevalence of anemia are only slightly different between women with and without VAR2CSA antibodies, thus this effect may not be biologically relevant.

With samples from a large cohort of pregnant women from Kilifi, VSA_PAM_ IgG levels were measured by flow cytometry against various *P. falciparum* placental isolates and compared to hemoglobin levels (57). Increased severity of maternal anemia was correlated with low IgG levels in chronic cases of PAM (placentas infected with parasites and hemozoin pigment present) and the level of VSA_PAM_ antibodies in these women was a strong predictor of maternal hemoglobin levels. However, no correlation was observed in women with acute or past placental infection.

### Studies That Inversely Correlate VSA_PAM_ Antibodies to Maternal Anemia

The findings from six studies suggest that VSA_PAM_ antibodies can reduce the severity of maternal anemia. In Malawi, two studies investigated the function of VSA_PAM_ antibodies by testing for opsonic phagocytosis. The first study revealed that a two-fold increase in the phagocytosis index was associated with a significant, 70% reduction in the odds of developing maternal anemia at delivery (51). Another study investigated third trimester serum samples and found that VSA_PAM_ IgG reactivity in flow correlated with activity in the opsonic phagocytosis assay (r = 0.60), but serum from anemic women displayed significantly lower opsonic activity (52). Notably, coinfection with HIV reduced opsonic activity, but did not affect inhibition of binding levels or total IgG against VSA_PAM_ (52).

In a later study from Malawi, antibody titres and function correlated to maternal hemoglobin at 36 weeks gestation, infant birth weight, gestational age, infant length, and placental histology at delivery (53). There was a significant positive association between IgG reactivity against CS2 parasites and maternal hemoglobin levels; a 10% increase in reactivity in flow corresponded to a hemoglobin increase of 0.5 g/L. Similarly, a 10% increase in the phagocytosis index corresponded to a hemoglobin increase of 0.4 g/L. Collectively, these studies support the hypothesis that opsonic phagocytosis could be an important effector mechanism of protection from maternal anemia.

VSA_PAM_ antibody-mediated protection from anemia may relate to the number of *var2csa* genes per *P. falciparum* genome in the parasites that infect pregnant women (54). Parasites with more than one *var2csa* gene are more common in PAM, compared to the non-pregnant population, and may provide a selective advantage over parasites with only one *var2csa* gene. In the Cameroonian village of Ngali II, increasing *var2csa* copy numbers in the parasites infecting pregnant women were associated with higher hemoglobin levels at delivery, but not with birth weight. However, in another region, Yaoundé, this relationship was reversed, with a negative correlation observed between *var2csa* copy number and birth weight. In Ngali II, IgG against DBL4ϵ, but not the full-length VAR2CSA, correlated positively with gene copy number. The authors hypothesized that parasites with multiple copies of *var2csa* may elicit immunity to specific DBL domains of VAR2CSA, and the protection observed might be due to a strong immune response that develops in women infected with these parasites.

Along with titre and functional assays, Chandrasiri et al. examined cytokine profiles of pregnant women from Sudan (55). Cytokine profiles were measured with a multiplex bead array that detected IL-1β, IL-6, IL-8, IL-10, IL-12, and TNFα. IFNγ was also measured by ELISA. Distinct cytokine profiles were observed in women with severe PAM compared to uninfected controls, with IFNγ, IL-6, and IL-10 significantly elevated. Parasite density was positively associated with levels of pro-inflammatory cytokines IL-6 and IL-8. Women with severe cases had low levels of IgG against VSA_PAM_ tested in flow, compared to uninfected controls. However, antibodies against DBL5ϵ were significantly greater than controls and negatively correlated with IL-1β, IL-6, and IL-8. Hemoglobin levels in these women were also negatively associated with IL-1β, IL-6, IL-8, and TNFα. Consistent with the studies from Malawi, opsonizing activity was positively associated with maternal hemoglobin at study enrollment. This study revealed associations between cytokine levels and VSA_PAM_ antibodies. An influx of pro-inflammatory cytokines could increase the risk of maternal anemia, but the presence of antibodies may have an effect on cytokine profiles.

Only one study from outside Africa identified a positive correlation between VSA_PAM_ antibodies and hemoglobin levels. This study was based in Colombia and examined a cohort of women with submicroscopic PAM (56). While VAR2CSA-specific antibody levels did not correlate with hemoglobin levels, the activity of these sera in the IBA was significantly associated with higher maternal hemoglobin levels at delivery. Interestingly, an association between antibody titre or function was not seen with birth weight, which is more commonly observed than associations with maternal anemia.

## Trends and Caveats

The results summarized above are divided on whether VSA_PAM_ antibodies contribute to protection from maternal anemia induced by PAM. These conclusions are consistent with a recent meta-analysis that examined associations between VSA_PAM_ antibodies and poor pregnancy outcomes, and included many of the same studies (58). Overall, the meta-analysis suggested that VSA_PAM_ antibodies are more likely to be markers of infection, instead of a source of protection against adverse outcomes, including anemia (58). However, additional analyses of the primary data provided by the authors revealed heterogeneous associations of VSA_PAM_ antibodies with anemia depending on a number of variables, such as gravidity, study design and the type of assay used to characterize antibodies. Considering these data and the results from the studies described previously, we identified various factors that may explain the discrepancies between these study findings.

### Study Setting

The level of malaria transmission in the study region influences the frequency of exposure to parasites and the diversity of strains. In turn, this could impact the magnitude and breadth of antibodies to VSA. Lloyd et al. collected samples from Yaoundé, a low transmission area with an entomological inoculation rate of approximately one infectious bite per person per month (49). In this study, just over half of women with a placental infection had antibodies to VAR2CSA, and this frequency was only 26.9% among multigravid women. Without sufficient exposure during pregnancy, a correlation between antibodies and pregnancy outcomes may not be detected. However, the study site of Fried et al. had intense seasonal transmission but there was no association between VSA_PAM_ antibodies and anemia, despite a positive correlation between infection history and antibody titres against several VAR2CSA subdomains (48). In this setting submicroscopic infections (SMI) were common, with a frequency between 19.8% and 25.4% in women who were blood smear negative at enrollment, and these infections were associated with an increase in VSA_PAM_ antibody levels, particularly in multigravid women. SMI may boost antibody responses without contributing to poor pregnancy outcomes, thus an association between VSA_PAM_ antibody titres and outcomes may not be observed. A similar finding was observed in Colombia, a low transmission setting, where history of SMI did not correlate directly with maternal hemoglobin measurements (56). Based on these few studies, it is unclear how transmission intensity impacts associations between VSA_PAM_ antibodies and anemia.

In certain settings, the fraction of anemia attributable to malaria may be low, masking a protective effect of VSA_PAM_ antibodies. In one of the cohorts from Malawi, 62% of women were anemic and the mean hemoglobin level was 10.6 g/dL, likely reflecting non-malarial causes of anemia (43). In Kenya, Staalsoe et al. also reported overall low hemoglobin values, with 15.6% of women having hemoglobin concentrations under 7 g/dL (57). Ndam et al. reported 62% of women were anemic at enrollment and 45% at delivery in a cohort from Benin, with only 16% and 12% of women testing positive for malaria infection at enrollment and delivery, respectively (41). Several factors besides malaria could influence maternal anemia, including micronutrient deficiency, coinfections, and quality of prenatal care [reviewed in (24, 25)]. Interestingly, in the cohort investigated by Chandrasiri et al. where there was a protective association between VSA_PAM_ antibodies and maternal anemia, women were given supplements (including iron) and the median hemoglobin was 11 g/dL (53). Perhaps this supplementation controlled for malaria-independent causes of anemia, revealing an association between the VSA_PAM_ antibodies and malaria-attributable anemia.

### Study Design

Depending on the study design, samples are collected at various times during pregnancy and/or at delivery and this could affect the interpretation of study findings. For example, VSA_PAM_ antibodies detected early in pregnancy may indicate a prior exposure to VSA_PAM_ in a previous pregnancy; thus, a pregnant woman may begin pregnancy with some immunity that may be boosted during subsequent pregnancies. Staalsoe et al. found that by six months postpartum, titres of VSA_PAM_ IgG decreased, but levels were boosted in women by the second trimester of their next pregnancy (59). Modeling studies estimate that VAR2CSA antibodies can persist over many years (60). Of those studies that reported an association between VSA_PAM_ antibodies and maternal hemoglobin levels, 4 out of 6 assessed antibody titre and/or function measured at first to second trimester (51, 53, 55, 56). In contrast, most of the studies that did not find a protective association with maternal anemia collected samples late in pregnancy, in the third trimester and at delivery (40, 41, 44, 46, 49, 45). If these VSA_PAM_ antibodies developed as a result of infection late in pregnancy, increased antibody levels may reflect recent boosting from infection and obscure a relationship between antibody and protection from anemia. Thus, VSA_PAM_ antibodies detected later in pregnancy may be markers of infection, rather than a source of protection, as noted by Cutts et al. (58), and others.

The gravidity of women included in the study can also greatly impact the titre, persistence and quality of antibodies investigated. Antibodies against VSA expressed in PAM develop with each successive infection in pregnancy; in high transmission settings, multigravid women are more likely to have antibodies that correlate with better pregnancy outcomes than primigravid women, supporting that these antibodies are maintained into the next pregnancy. Fried et al. reported that VSA_PAM_ antibodies from primigravid women are short-lived, compared to those from secundigravid and multigravid women (48). In the 2010 Ataide et al. study, only primigravid women were included and samples were collected in the late third trimester, with no protective association between VSA_PAM_ antibodies and anemia observed (42). The same cohort was followed into the second pregnancy, and again there was no association between maternal hemoglobin and VSA_PAM_ IgG or opsonic phagocytosis activity (43). Thus, protective qualities of VSA_PAM_ antibodies might not fully develop until infections in multiple pregnancies have occurred. In Malawi, recognition of iRBCs by VSA_PAM_ antibodies from secundigravid and multigravid women, but not primigravid women, was associated with a reduced odds of anemia (47, 58). In contrast to the related studies discussed above, this association was observed in samples collected in the third trimester of pregnancy.

Another important consideration is treatment history. IPTp with 1500/75 mg of sulphadoxine and pyrimethamine (SP) during antenatal care visits is recommended by WHO in most areas in sub-Saharan Africa to reduce placental malaria (61). This treatment has the potential to affect the acquisition of VSA_PAM_ antibodies, as parasitemia is prevented or cleared to the point that plasma cells will not produce antibodies against VSA_PAM_ targets. In Ndam et al. where no association was observed between VSA_PAM_ antibodies and maternal anemia at delivery, women received two doses of SP during pregnancy (41). Aitken et al. reported lower levels of VSA_PAM_ antibodies associated with IPTp treatment (47). Similar observations were made in other studies from Kenya (50) and Ghana (62), where reductions in antibody titres were associated with IPTp treatment (though pregnancy outcomes were not reported). In contrast, Serra-Casas et al. failed to observe an effect of IPTp on VSA_PAM_ antibody levels (46). Therefore, the effects of IPTp on VSA_PAM_ antibodies in relation to anemia are unclear. But, it is unlikely that IPTp treatment contributes to the severity of maternal anemia itself, as a controlled or lowered parasitemia should benefit the health of the mother.

Finally, HIV is known to affect the humoral response in PAM, which was observed by a number of studies examined in this review (42–44, 46, 52). In the two studies by Ataide et al. VSA_PAM_ phagocytic antibodies were reduced in HIV positive women compared to women who were HIV negative but this was only observed when antibody activity was measured using a functional assay; there was no change in the antibody reactivity to iRBCs by flow cytometry (42, 43). The presence of HIV in a cohort may therefore reduce VSA_PAM_ antibody levels and/or function to a level too low to improve outcomes, particularly in lower transmission settings (49) or when women are treated with IPTp (46).

## Assay Selection

It is necessary to assess antibody titre and particularly function to make comparisons with pregnancy outcomes. From the group of studies that did not find an association with maternal anemia, IBA was the primary functional assay used, with only two studies in this group that measured opsonic phagocytosis activity (42, 43). Inhibition of adhesion by VSA_PAM_ antibodies is more commonly associated with higher infant birth weight due to *P. falciparum* infections during pregnancy (40). In only one study, from Colombia, adhesion-blocking activity was associated with increased maternal hemoglobin at delivery (56). Several of the studies point to opsonic phagocytic activity as a particularly important correlate of protection against maternal anemia. Chandrasiri et al. showed that only antibodies with opsonic phagocytic activity were associated with reduced odds of severe malaria, rather than antibody titre measured by ELISA (55). Similarly, Feng et al. observed a correlation between a reduced odds ratio of maternal anemia and VSA_PAM_ antibody activity both by flow cytometry and opsonic phagocytosis, but the relationship between phagocytic activity was stronger than VSA_PAM_ seroreactivity in flow (51). Interestingly, in one study, VSA_PAM_ IgG measured by flow correlated more strongly with opsonic activity than anti-adhesion activity in the IBA (52).

Functional assays can also focus on specific IgG subclasses, rather than measuring total IgG by flow cytometry or ELISA. IgG1 and IgG3 are cytophilic subclasses that interact with Fc receptors on other immune cells, including monocytes and macrophages, and promote opsonic phagocytosis (63). These are the dominant subclasses in PAM, while IgG2 and IgG4 levels do not significantly change (64). Opsonic phagocytosis assays specifically measure these cytophilic subclasses, whereas testing for total IgG may not reveal a relationship with pregnancy outcomes. Future studies should characterize antibody populations using functional assays, along with assessments of titre and subclass, to test for associations with anemia.

Overall, there are many factors that can affect the relationship between maternal anemia and VSA_PAM_ antibodies. Based on our interpretation of the studies described above, the discordance between the study findings may be attributed in part to the timing of sample collection and assay selection.

## Potential Mechanisms of Antibody-Mediated Protection from Maternal Anemia in PAM

Though evidence for a specific role for VSA_PAM_ antibodies in protection against maternal anemia is currently inconclusive, we can speculate on putative mechanisms of protection to help inform future research (**Figure 1**). We hypothesize that protective antibodies could directly target the iRBCs in the placental environment, either through opsonization or by physically blocking adhesion to CSA. Based on the group of studies that found an association with improved outcomes, VSA_PAM_ antibodies that mediate opsonic phagocytosis of iRBCs may be more likely to be protective against maternal anemia than antibodies that inhibit parasite binding to the placenta, suggesting these antibodies may have distinct effector mechanisms. Antibodies with dual functions may also exist.

Antibody binding to iRBCs can lead to different downstream effects. Clearing the iRBC from the placenta either by preventing sequestration and/or recruiting immune cells would prevent the hemolysis associated with anemia and the associated inflammatory response. Erythropoiesis is also susceptible to a dysregulated pro-inflammatory response, a common cause of anemia in uncomplicated malaria and PAM [reviewed in (24)]. VSA_PAM_ antibodies can potentially contribute to these responses by reducing cytokine dysregulation and targeting immune cells to iRBCs. As observed by Chandrasiri et al. pro-inflammatory cytokines IL-1β, IL-6, and IL-8 negatively correlated with antibody titres against DBL5ϵ in pregnant women with severe malaria (55). In this study, opsonizing antibody activity and cytokine profiles were not compared directly, but maternal hemoglobin levels increased with increasing levels of opsonizing antibodies (53). In another study, cytokine secretions were measured after opsonic phagocytosis of CS2 parasites had occurred, using serum from Malawian pregnant women. These results were compared to antibody-independent phagocytosis and a shift in the cytokine profile was observed, with an increase in production of IL-1β and TNFα when antibodies were present (65). Opsonized iRBCs activated the inflammasome and production of IL-1β by macrophages, but this was also observed with unopsonized iRBCs. Thus, the secretion of pro-inflammatory mediators likely occurs in the placenta with and without sufficient titres of VSA_PAM_ antibodies. Perhaps the labeling of iRBCs with VSA_PAM_ antibodies leads to Th1-like immune responses to appropriately respond to the parasite without leading to dysregulated inflammation and inhibition of erythropoiesis.

### Future Directions

As the pathways leading to anemia in PAM are complicated and multifactorial, further research is critical to better understand any potential underlying immune mechanisms in response to malaria infection that contribute to or protect women from anemia. Longitudinal studies should be continued in and outside of Africa to increase the body of knowledge available on infection, VSA_PAM_ antibody levels, and pregnancy outcomes. Studies should be conducted in many diverse settings to reveal context-specific effects on the acquisition of PAM immunity and the prevalence of maternal anemia. Treatment practices and circulation of other pathogens in a particular area can also affect humoral immunity.

In those studies where VSA_PAM_ antibodies are associated with higher hemoglobin levels in mothers, it will be exciting to unravel the underlying mechanisms of protection. We propose a basic model for how these antibodies that target placental iRBCs could block downstream pathogenic effects that lead to anemia, yet the mechanisms that directly contribute to this process need to be delineated. While the data support a role for these antibodies in blocking sequestration in the placenta and promoting opsonic phagocytosis, emerging evidence linking the innate and adaptive immune systems merit exploration. For example, data is emerging on the role of the complement system in uncomplicated malaria. The antibody-mediated activation of the classical complement pathway was observed in samples from children and was an indicator of protective immunity (66). In addition, NK cells may participate in the response to PAM. Long et al. showed that antibodies from VAR2CSA-immunized rabbits induced iRBC lysis *in vitro* (67). Further, human IgG pooled from plasma of multigravid women and incubated with parasites and NK cells inhibited parasite growth by approximately 50%. No connection to anemia was examined, but this is an interesting component of the PAM response that should be further investigated with respect to pregnancy outcomes.

This review highlights the complexity of the body’s response to PAM and the large gaps in the current knowledge on immune mechanisms underlying protection. Pregnant women and young children remain at great risk of morbidity and mortality from malaria. Continued research in this area will add to our understanding of PAM and potentially reveal novel therapy and vaccine strategies to combat this disease.

## Author Contributions

MCW conducted the literature review. MCW and SKY jointly wrote the manuscript. All authors contributed to the article and approved the submitted version.

## Funding

MCW holds a CIHR Frederick Banting and Charles Best Canada Graduate Scholarship.

## Conflict of Interest

The authors declare that the research was conducted in the absence of any commercial or financial relationships that could be construed as a potential conflict of interest.

The handling editor declared a past co-authorship with one of the authors, SY.
